# Alpha oscillations related to self-other integration and distinction during live orchestral performance: A naturalistic case study

**DOI:** 10.1177/03057356221091313

**Published:** 2022-04-29

**Authors:** Justin Christensen, Lauren Slavik, Jennifer J Nicol, Janeen D Loehr

**Affiliations:** 1 Department of Psychology, University of Saskatchewan, Saskatoon, Canada; 2 Department of Educational Psychology and Special Education, University of Saskatchewan, Saskatoon, Canada

**Keywords:** ensemble music performance, naturalistic case study, self-other integration and distinction, alpha oscillations, EEG, joint action

## Abstract

Ensemble music performance requires musicians to achieve precise interpersonal coordination while maintaining autonomous control over their own actions. To do so, musicians dynamically shift between integrating other performers’ actions into their own action plans and maintaining a distinction between their own and others’ actions. Research in laboratory settings has shown that this dynamic process of self-other integration and distinction is indexed by sensorimotor alpha oscillations. The purpose of the current descriptive case study was to examine oscillations related to self-other integration and distinction in a naturalistic performance context. We measured alpha activity from four violinists during a concert hall performance of a 60-musician orchestra. We selected a musical piece from the orchestra’s repertoire and, before analyzing alpha activity, performed a score analysis to divide the piece into sections that were expected to strongly promote self-other integration and distinction. In line with previous laboratory findings, performers showed suppressed and enhanced alpha activity during musical sections that promoted self-other integration and distinction, respectively. The current study thus provides preliminary evidence that findings from carefully controlled laboratory experiments generalize to complex real-world performance. Its findings also suggest directions for future research and potential applications of interest to musicians, music educators, and music therapists.

Ensemble music performance demands continuous adaptation and alignment between performers to allow for the expression of shared communicative goals. Researchers have shown that people participating in complex coordination tasks engage in continuous sensorimotor coupling to incoming perceptual information while also maintaining a degree of separation between themselves and their co-agents (e.g., Fairhurst et al., 2019; MacRitchie et al., 2018; Varlet et al., 2019). This allows them to achieve precise interpersonal coordination while still having autonomous action control. Ensemble musicians in particular maintain an awareness of the relationship between their part and the whole when performing together, dividing their attention between their own part, others’ parts, and the overall sound (e.g., Keller, 2008, 2013; Loehr et al., 2013). Moreover, they dynamically shift the degree to which others’ actions are integrated with their own or kept distinct from their own, depending on how beneficial others’ actions are for their ability to synchronize and to contribute to the overall sound at any given moment (e.g., Fairhurst et al., 2019; Novembre et al., 2016; Uhlig et al., 2013). Past research in a laboratory setting has suggested that alpha oscillations in sensorimotor brain regions index this dynamically changing process of integration and distinction (Novembre et al., 2016). To further our understanding of how musical behaviors are implemented in the brain, the current study investigated whether the same neural underpinnings of integration and distinction would be evident in a naturalistic performance setting, that is, during a live performance of a complete musical piece.

## Self-other integration and distinction

Complex coordination tasks like ensemble music performance demand a careful balance between integrating others’ actions into one’s own action planning and execution, termed self-other integration, and maintaining a distinction between one’s own and others’ actions, termed self-other distinction (Fairhurst et al., 2019). Self-other integration is thought to arise from close links between perception and action. It can emerge spontaneously as a result of perception-action coupling, such as when walkers spontaneously synchronize their speeds and gaits (van Ulzen et al., 2008). It can also occur when people form cognitive representations of others’ goals and actions. For example, people often integrate others’ actions into their own action planning and execution quasi-automatically, even when it would benefit their performance to ignore others’ actions (e.g., Sebanz et al., 2003). When people pursue shared goals together, self-other integration can help them anticipate and adjust to each other’s actions (Knoblich et al., 2011).

Self-other integration may be especially likely when people share goals and perceive their actions to be interdependently linked with the actions of their co-performers (Iani et al., 2011). Music ensembles spend a considerable amount of their rehearsal time negotiating their shared performance goals, forming idealized representations of the conductor’s performance intentions, and integrating each other’s movement patterns into their action plans. That is, they work hard to develop a strong interdependent relation of actions and action plans. As a result, there may be times when musicians find it difficult to avoid synchronizing with one another in performance (Keller et al., 2014; MacRitchie et al., 2018; Nowicki et al., 2013). However, even if self-other integration can be quasi-automatic and difficult to resist, long rehearsals of negotiating shared intentions also suggest that self-other integration can be effortful (Keller et al., 2016). This can be seen in performers not only increasing the size and regularity of their movements to enhance coordination (Goebl & Palmer, 2009; Vesper et al., 2011, 2017) but also in their development of ancillary movements, such as body sway, to facilitate the communication of expressive intentions (Chang et al., 2017, 2019; Keller et al., 2016).

Despite sharing the goal of creating a unified sound, and working toward integrated representations of each other’s actions, each performer’s primary responsibility is nevertheless to properly play their own part (Keller et al., 2016). To achieve this, performers need to maintain a sense of autonomous action control, which is accomplished by maintaining a degree of distinction between themselves and their fellow performers (Fairhurst et al., 2019; Keller et al., 2016; Pacherie, 2012). For instance, if singers are unable to hear their own voice when singing in a choir, their vocal control suffers (Ternström, 1999). Similarly, pianists respond slower and make more errors when they hear musical sounds incongruent with their intended actions during action planning, often altering their action to match that of the sound distractor (Drost et al., 2005a, 2005b) Performers therefore develop the ability to enhance representations of their own actions and/or selectively inhibit representations of other ensemble members’ actions when needed (Bigand et al., 2000; Keller et al., 2016; Liepelt et al., 2016). Self-other distinction is especially likely when a musician’s instrumental timbre is similar to their co-performers’ and they have difficulty separating their sounds from co-performers’ due to acoustic masking (Fairhurst et al., 2019; Meyer, 2009). Self-other distinction also occurs when an individual’s intended actions are incompatible with the activity surrounding them (Novembre et al., 2016).

## Neural correlates of self-other integration and distinction

Recent laboratory findings suggest that the processes of self-other integration and distinction during joint music performance may be indexed by cortical alpha oscillations (Novembre et al., 2016). In other words, cortical alpha oscillations might reflect the implementation of self-other integration and distinction at the neural level (which in turn might result from performers’ conscious intentions for their performance, their cognitive representations of the musical piece, perception-action coupling between their own and others’ actions, or some combination thereof; see Pacherie, 2012, for further discussion of how different intentional levels are integrated within and between performers in joint actions such as ensemble music performance). Our focus in the current study was on the neural implementation level because examining performers’ brain activity offers the possibility of illuminating dynamic changes in self-other integration and distinction that occur as a performance unfolds. Examining neurophysiological data also complements other research strategies such as analyzing performers’ verbal and nonverbal behavior during performances or asking performers to reflect on their coordination strategies after a performance (Deschepper et al., 2017; Ormerod & Ball, 2017). For example, performers’ brain activity can reveal processes that might not be consciously accessible for verbal reports or observable in nonverbal behavior, as well as processes that occur on a relatively short timescale (e.g., within bars or sections of a musical piece). On a broader level, advancing knowledge about how musical behavior is implemented at the neural level can inform practices related to music performance, education, and therapy (e.g., Collins, 2013; Legge, 2015).

How might cortical alpha oscillations reflect self-other integration versus distinction? On the one hand, *increases* in alpha activity are thought to index processes related to self-other *distinction*. For example, one of the most prominent theories on alpha oscillations, gating by inhibition (Jensen & Mazaheri, 2010), posits that increases in the amplitude of alpha oscillations index an inhibitory process that blocks out distracting sensory input and irrelevant downstream processing. Consistent with this theory, increases in alpha activity occur during the inhibition of either visual (Scheeringa et al., 2009) or auditory distractors (van Dijk et al., 2010) during working memory tasks that focus on retrieving internal representations. Increases in alpha activity also occur when people have to block out distracting noise to focus on a single voice in a noisy environment (Strauß et al., 2014). Similarly, distractor objects in one-half of the visual field increase alpha activity over the contralateral hemisphere in divided vision tasks (Zumer et al., 2014).

On the other hand, *suppressed* alpha activity over central and parietal scalp locations may index the activation of perception-action coupling (Fox et al., 2016), which is thought to be linked to self-other *integration*. Consistent with this hypothesis, alpha suppression occurs at similar scalp locations both when a person executes an action and when they observe others executing the same action (Babiloni et al., 2002, 2011; Kuhlman, 1978; Muthukumaraswamy & Johnson, 2004; Muthukumaraswamy et al., 2004; Nuara, 2020). Furthermore, social activity, especially that which is coordinated and/or is directed at the participant, increases alpha suppression. For instance, Tognoli and colleagues (2007) asked pairs of participants to move their fingers at their own pace, allowing the participants to spontaneously couple the speed of their finger movements. A form of alpha suppression (labeled phi_2_) increased in tandem with this coupling process. Naeem and colleagues (2012) had pairs of participants move their fingers, either while ignoring each other, moving in phase with one another, or moving in antiphase with one another. Participants who ignored each other while moving their fingers showed the least amount of alpha suppression, while those moving in antiphase with one another showed the strongest alpha suppression. Konvalinka and colleagues (2014) had participants tap either interactively with another participant or with a nonresponsive computer. Participants interacting with one another showed greater alpha suppression than those interacting with a nonresponsive computer. Other aspects of social context also modulate alpha suppression, such as personal involvement in the task (Perry et al., 2011) or having a social history with others involved in the task (Kourtis et al., 2013).

Alpha activity also indexes self-other integration and distinction specifically in the context of joint music performance. Novembre and colleagues (2016) had pianists play duets by performing one hand of several two-handed Bach chorales. They performed the first phrase of the chorale together at the same tempo, and then modulated to a new tempo, either making a congruent tempo change (both moving to a slower or faster tempo) or an incongruent tempo change (one partner moved to a faster tempo while the other moved to a slower tempo). Incongruent tempo changes, which promoted self-other distinction, increased alpha activity; congruent tempo changes, which promoted self-other integration, suppressed alpha activity. Further indirect support for the idea that alpha suppression indexes self-other integration during joint music performance comes from a recent study by Vanzella and colleagues (2019). They recorded brain activity from pairs of violinists while they played canonic duets. Violinists showed higher levels of oxygenated hemoglobin in sensorimotor brain areas (a different measure of brain activity that correlates with motor-related alpha suppression; Lachert et al., 2017) when they performed an accompanying role, which promotes self-other integration due to heightened processing of external sensory information, compared to when they performed the leading role or performed alone.

## The current study

The purpose of the current study was to examine alpha oscillations related to self-other integration and distinction during ensemble music performance in a naturalistic performance context. Our primary goal was to investigate whether patterns of EEG alpha activity previously reported in a controlled laboratory experiment (Novembre et al., 2016) would also be evident among members of a large orchestra performing a repertoire piece under naturalistic conditions. Although naturalistic contexts for data collection are represented in music psychology research (e.g., Beck & Rieser, 2022; Forrester, 2010; Jakubowski & Ghosh, 2021), naturalistic studies involving EEG data are less common, despite the importance of naturalistic contexts for direct observation of individuals in their natural setting and for supporting the ecological validity of laboratory research (among other things; Andrade, 2018; Hoc, 2001). Furthermore, although some studies show that interpersonal coordination mechanisms generalize from relatively simple small-scale interactions in controlled laboratory settings (e.g., synchronized finger tapping) to larger-scale interactions under naturalistic conditions (e.g., ensemble music performance; Repp & Su, 2013), findings established under controlled laboratory settings do not always generalize to naturalistic settings that entail more complexity or additional cognitive or situational constraints (e.g., Kingstone et al., 2008; Shamay-Tsoory & Mendelsohn, 2019). As a result, it is important to investigate research questions across a variety of settings that are representative of the contexts in which the phenomenon of interest arises (Holleman et al., 2020; Nastase et al., 2020). We therefore carried out a case study in which we undertook a descriptive analysis of four performers’ alpha activity during a single instance of live orchestral performance of a complete musical piece. Specifically, we measured alpha activity from four violinists during a live orchestra performance in a concert hall setting, while they performed a musical piece selected from their orchestral repertoire. Before analyzing the alpha activity, we performed a score analysis to divide the musical piece into sections that were expected to promote self-other integration and distinction. We predicted that sections promoting self-other integration and distinction would elicit alpha suppression and enhancement, respectively.

## Methods

### Data collection context

EEG data were recorded from four violinists during an orchestra performance of Derek Charke’s *Élan*. The performance took place at the beginning of a concert dress rehearsal, in the orchestra’s primary home performance hall, specifically for data collection. The musical piece was selected from the orchestra’s active repertoire (i.e., from pieces they had performed within approximately a year before data collection took place). *Élan* was chosen because it is a challenging contemporary work with a relatively short duration (approximately 2 min) that contains musical sections that were expected to strongly promote self-other integration and distinction, respectively, as described in the Score Analysis and Predictions section below. The piece was performed in a single take without prior rehearsal on the day of data collection. The conductor consulted with the research team about directions for the performance prior to data collection. Ethical approval was obtained from the university’s institutional review board before participant recruitment.

### Participants

Four right-handed violinists (2 females, mean age = 36, mean years of violin experience = 31) were recruited from a mid-sized (approximately 60 members) Canadian professional symphony orchestra that performs approximately 20 concerts per season and regularly invites acclaimed soloists from across Canada and the world. We recruited violinists as participants because professional violinists produce relatively little EMG activation from their arm movements and minimal face and head movements when they play their instruments, which could otherwise contaminate EEG recordings (Bazanova et al., 2003; Goncharova et al., 2003). The sample size of four was determined by the number of EEG caps available. We recruited two violinists from the *first violin* section and two from the *second violin* section. The first and second violin sections often take on different musical roles; specifically, the first violin section very often plays the melody, whereas the second violin section often harmonically and rhythmically supports the melody. These differing musical roles influenced our predictions for alpha activity in the canon section (see the section “Score analysis and predictions”). To sample the range of performers within the violin section, we also recruited one *principal player* and one *section player* from each section. The principal player typically takes on a leadership role relative to the other players in their section (taking the orchestral solos and choosing the bowing for the section in advance), while the section players work to support the leader of their section. All participants provided written informed consent before beginning the study.

### Score analysis and predictions

**Figure 1.**
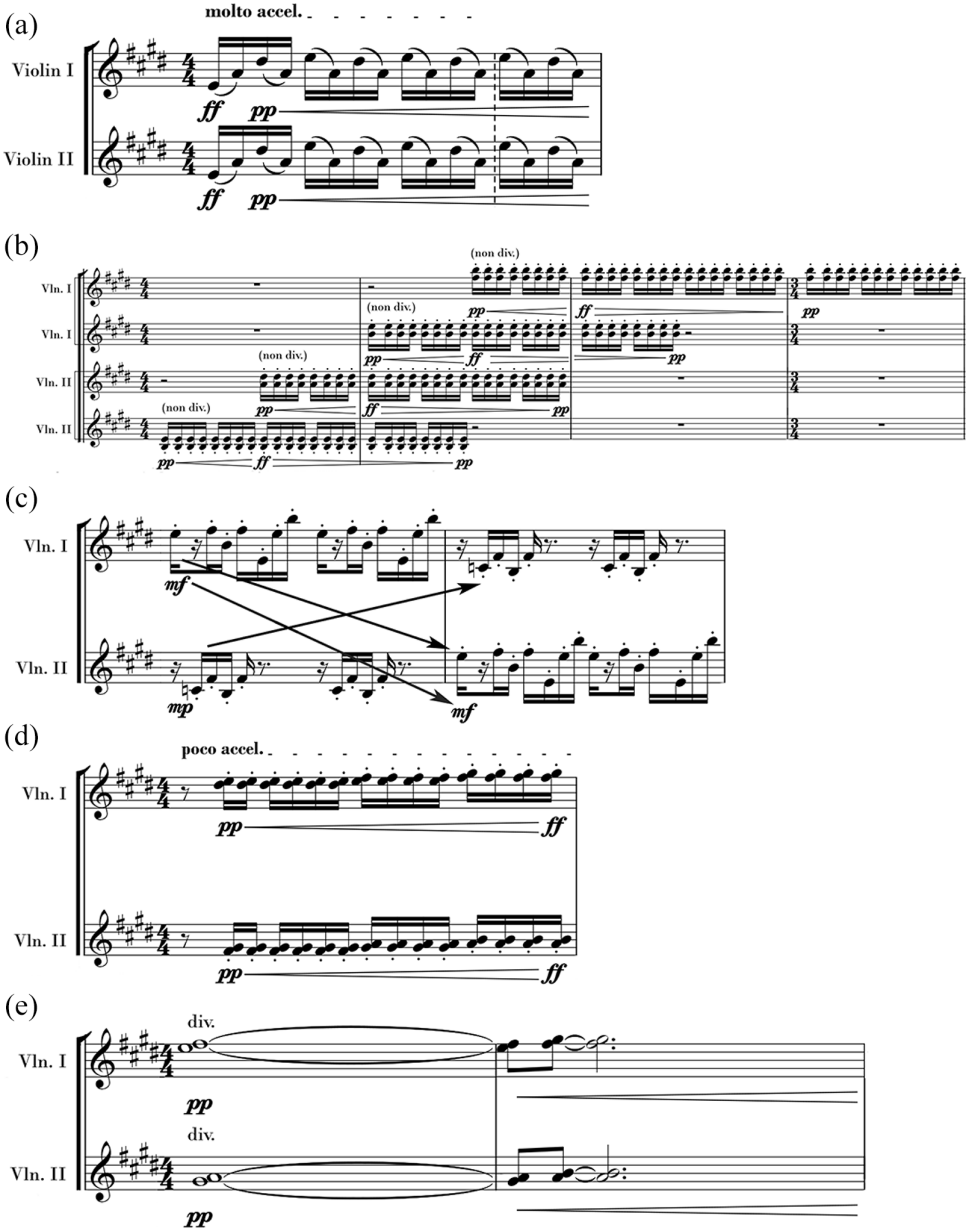
Examples of the unison (a), polyphonic (b), canon (c; arrows indicate a passing back and forth of the melody and accompaniment between the first and second violins), homo-divisi (d), and slow homorhythmic (e) section types. Score examples used by permission of © 2016 Derek Charke—www.charke.com.

Prior to EEG analysis, the first author^1^ undertook a score analysis and identified five musical section types. Illustrative examples of the first and second violin parts within each section type are shown in Figure 1. The top panel of Figure 1 shows an example of a *unison* section. Unison sections required not only the four recorded violinists but also the entire orchestra to perform their pitches, rhythms, and smooth tempo modulations in unison with one another (with the exception that some performers doubled the melody at the octave while others only supported this unison by playing some of the notes). Unison sections were expected to strongly promote self-other integration, because the performers produced highly congruent actions with the goal of remaining in unison (MacRitchie et al., 2018). We therefore predicted that all performers would display alpha suppression during unison sections.

The second panel of Figure 1 shows an example of a *polyphonic and polyrhythmic divisi* section (henceforth referred to as the *polyphonic* section type). In this section type, the four violinists entered at different times, and performed different phrasing, dynamics, and pairs of notes than the violinist sitting next to them.^2^ The polyphonic section type included a cascade of violin entries spaced one bar apart, while syncopated rhythms in the clarinets and oboes and sporadic entries of triplets and dotted eighths in the basses and percussion challenged the violins’ rhythmic character. The polyphonic sections were expected to strongly promote self-other distinction because the performers produced highly incongruent actions from one another and would therefore be compelled to inhibit distracting sensory input from the performers around them. We therefore predicted that all four performers would display alpha enhancement during this section type.

The third panel of Figure 1 shows an example of the *canon* section. The canon section had a melody of two bars in length. The melody started in the first violin section and was imitated by the second violin section after the first bar. The rhythms of the accompanying part played by the second violins were complementary to the melody played by the first violins; that is, they filled in gaps in the melody played by the first violins. We expected that the canon section would generate a leader–follower dynamic, with the first violins taking the role of leader and the second violins taking the role of follower. This expectation was partly based on the convention that first violins more often take on a leading role while second violins take a supporting role (Adler, 2002), and partly based on previous research showing that when pairs of people tap in alternation (e.g., Bolt et al., 2016; Fairhurst et al., 2014; Wegner & Sparrow, 2007) and when violinists play in canon (e.g., Vanzella et al., 2019), a leader–follower dynamic emerges in which the first person to act takes on the role of leader and the other the role of follower. It also aligns with historical precedent, whereby in a canon, the initial melody is referred to as the *dux*, meaning leader, and the imitative melody is referred to as the *comes*, meaning follower (Walker, 2001). We further expected that the roles of leader and follower would promote self-other distinction and integration, respectively, because the role of leader in a joint action has been associated with a reliance on internal models while following has been associated with externally oriented processing (Fairhurst et al., 2014; Keller et al., 2016; Vanzella et al., 2019). We therefore expected the first violinists to display alpha enhancement and the second violinists to display alpha suppression in the canon section.

The last two panels of Figure 1 show the remaining two musical section types, which we did not expect to strongly promote either self-other integration or distinction.^3^ The fourth panel of Figure 1 shows an example of a *homorhythmic, divisi-pitch* section (henceforth referred to as the *homo-divisi* section type). In this section type, each violinist played a different note than the violinist sitting next to them, and the first violin section played different notes than the second violin section. However, the violinists still played the same rhythms, phrasing, and dynamics as one another. Additionally, the role of the violins in this section was to play interjections as part of a larger mixed texture (i.e., they played different notes, phrasing, and dynamics than most of the other instruments in the orchestra). Thus, this section contained qualities of both the unison and the polyphonic sections, and we did not have *a priori* predictions for the performers’ alpha activity in this section type.

The last panel of Figure 1 shows an example of a *slow homorhythmic* section. In this section type, the violins were part of a slow, homorhythmic, syncopated background texture along with the low strings and low brass. A running 16th-note melody was performed by the bassoons and violas. Each of the violinists played a different note from the violinist sitting next to them, contradicting the rhythmic unison created by more than half of the orchestra playing in rhythmic unison with one another. As this section was slow, syncopated, and had some characteristics of both the unison and polyphonic sections, we also did not have *a priori* predictions for the performers’ alpha activity in this section type.

### Procedure

The four participants were fitted with EEG caps just before the orchestra’s dress rehearsal began, in a separate room from the rest of the orchestra performers. The four participants were then seated in their usual seats within the orchestra. Laptops were placed on the floor next to the participants’ chairs to record the EEG data. EEG data were recorded during the orchestra’s performance of the musical piece and during 2 min of rest before and after the performance, during which time the participants sat quietly with their eyes open. The periods of eyes-open rest were used to determine the participants’ individual alpha frequencies (Corcoran et al., 2018; see the section “Data analysis”). At the beginning and end of each section (rest-before, performance, and rest-after), the participants were instructed to blink their eyes four times in synchrony with the conductor’s beat, at a rate of 80 bpm. These blinks were used to epoch the participants’ EEG data after the fact using the eye movements evident in the EEG data along with the time-coded video recording of the performance (Bœkgaard et al., 2014; Dimigen et al., 2011), as described in the sections “Data acquisition” and “Data processing”. After the second rest period, the EEG caps were removed from the participants’ heads and the orchestra carried on with their dress rehearsal.

### Data acquisition

EEG was recorded continuously from the participants using four separate LiveAmp amplifiers with 16 ActiCap Ag/AgCl active electrodes per participant (Brain Products GmbH, Germany). Electrodes were arranged according to the 10–20 system at FP1, FP2, F7, F3, FZ, F4, F8, FCZ, C3, CZ, C4, P3, PZ, P4, O1, and O2, using carefully positioned nylon caps. All electrodes were referenced to the right mastoid during recording. Impedance was kept below 20 kΩ. EEG signals were amplified within a band-width of 0.01–125 Hz and digitized with a sampling frequency of 500 Hz. Data were recorded using Brain Vision Recorder (ver. 1.21; Brain Products GmbH, Germany). Simultaneous video and audio were recorded with a Panasonic Lumix DMC-GH4 at 60 frames/s, with time and date burned into the image.

### Data processing

EEG data processing was performed offline using EEGlab v14.1.2 (Delorme & Makeig, 2004) and FieldTrip v20210507 (Oostenveld et al., 2011) in Matlab R2018a. First, the EEG data were epoched into rest-before, performance, and rest-after sections. The beginning of each section was marked by the eye-closing peak in the EEG data from the last blink before the section began. Likewise, the end of each section was marked by the eye-closing peak in the EEG data from the first blink after the section ended. Next, onset times for each musical section type were determined from the audio-video recording of the performance. An event marker was added to the EEG data for each musical section onset, based on elapsed time since the beginning of the performance epoch (i.e., the last blink before the performance section began^4^). The raw EEG data were then high-pass (pass-band: 1 Hz, −6 dB cutoff: 0.5 Hz) and low-pass (pass-band: 40 Hz, −6 dB cutoff: 45 Hz) filtered using a finite impulse response filter. An Adaptive Mixture Independent Component Analysis (AMICA) was performed and components reflecting blinks and eye movements were subtracted from the data. EEG data were then separated into 1.5 s epochs (0.5 s overlap) for the performance section and into 3 s epochs (1 s overlap) for the rest-before and rest-after sections. Additional artifacts were automatically detected and manually removed from the data using artifact rejection criteria that examined scalp-recorded voltage fluctuations (specifically: a peak-to-peak test with a 400-ms window width, 10-ms step size, and 125-µV threshold for sudden voltage shifts; a step function with a 200-ms window width, 10-ms step size, and 50-µV threshold for eye-blinks; and a step function with a 400-ms window width, 10-ms step size, and 25-µV threshold for horizontal eye movements). In addition, two electrodes from one participant were interpolated using a spherical spline because the electrodes showed large amounts of noise due to poor scalp-electrode contact during recording. Overall, 13.5% of epochs were rejected due to artifacts, leaving on average 28, 17, 12, 10, and 7 epochs per participant in the unison, polyphonic, canon, homo-divisi, and slow homorhythmic sections, respectively.

### Data analysis

Data analysis followed similar steps to those reported by Novembre et al. (2016). As in their analysis, we used the “mtmconvol” method in Fieldtrip with a window of five cycles and a window slide of 100 ms to perform a multitaper Hanning-windowed fast Fourier transformation on all artifact-free epochs, and we calculated power estimates for frequencies between 2 and 40 Hz in 1 Hz bins. We calculated participants’ individual alpha bands by using the restingIAF MATLAB function (Corcoran et al., 2018) to calculate center of gravity (CoG) values during the four minutes of rest from the C3, CZ, C4, P3, PZ, and P4 electrodes, and we defined individual alpha bands as ±2 Hz around each individual CoG. This yielded alpha bands of 7.9–11.9, 7.3–11.3, 8.3–12.3, and 7.1–11.1 Hz for the four participants, respectively.^5^ We then calculated alpha power during the musical performance within each participant’s alpha band at electrodes C3, CZ, C4, P3, PZ, and P4. We limited our analysis of alpha activity during the musical performance to centroparietal electrodes because Novembre et al. (2016) reported alpha enhancement and suppression at centroparietal electrodes only. Finally, we calculated normalized alpha power following the same calculations used by Novembre and colleagues (2016). Specifically, the average band power across conditions (c) was subtracted from each individual condition-specific band power, separately for each combination of electrode (e), frequency bin (f), and time bin (t). These difference values were then scaled by dividing each difference value by the average band power. Positive values of normalized alpha power therefore indicate a relative enhancement of alpha power in a given condition, and negative values indicate a relative suppression of alpha power.

$${\hat{P}}_{c(e,f,t)}=\frac{P_{c\left(e,f,t\right)}-\frac{1}{N}\times \sum_{C=1}^{N}P_{c(e,f,t)}}{\frac{1}{N}\times \sum_{C=1}^{N}P_{c(e,f,t)}}$$

Because we employed a descriptive case study design, our goal was not to conduct inferential statistical tests but rather to report descriptive statistics showing the normalized alpha power separately for each participant in each musical section type. In the section “Results”, we therefore plot normalized alpha power values separately for each performer. For ease of visual comparison with other EEG datasets that average across participants, we also plot the overall mean and *SD* across participants for the musical section types for which we had *a priori* predictions. We report the mean and *SD* across all four performers for the unison and polyphonic section types, in which the performers were expected to uniformly show alpha suppression or enhancement, respectively. We report the mean and *SD* separately for the first and second violinists for the canon section type, because we expected alpha activity to differ by the performer type. We do not report means across participants for the other two musical section types, because we did not have *a priori* reasons to choose which performers to average across.

## Results

**Figure 2.**
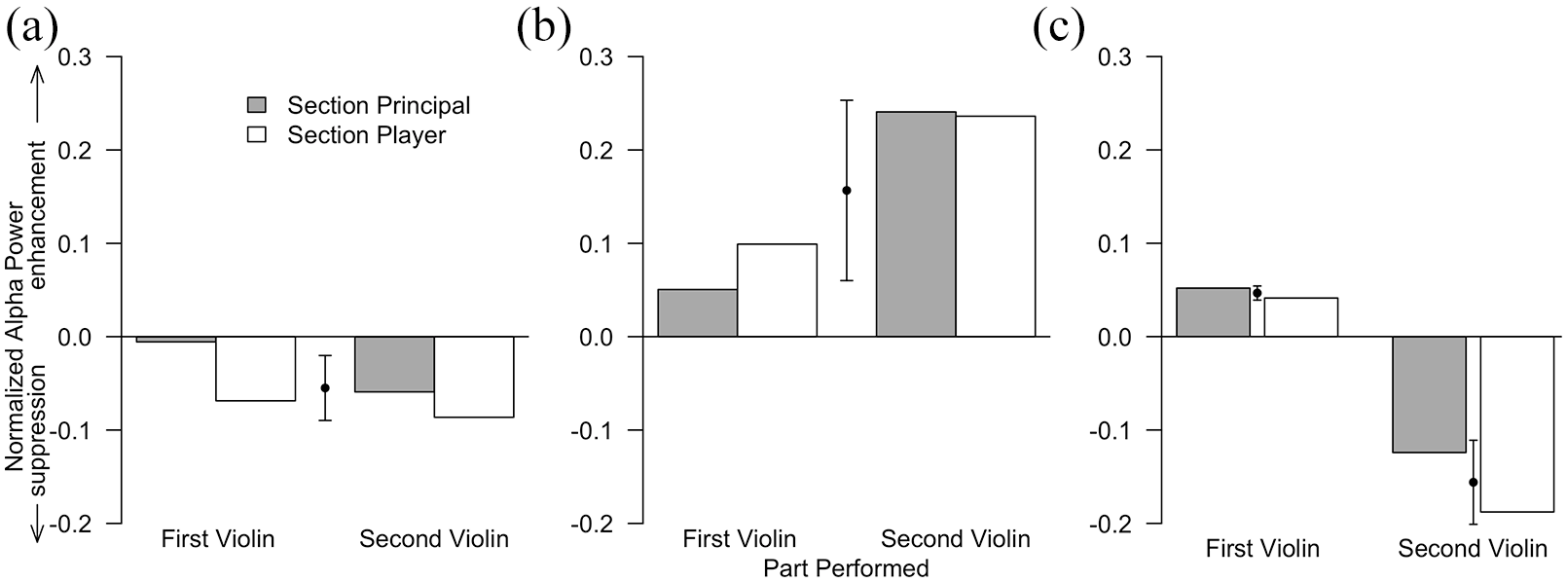
Normalized alpha power for each performer in the first and second violin sections in the unison (a), polyphonic (b), and canon (c) sections. Solid circles indicate the mean normalized alpha power (±*SD*) across performers.

Figure 2 shows the normalized alpha power for each performer, and averaged across performers, in the three musical section types for which we had *a priori* predictions about alpha activity. As shown on the ordinate axis in Figure 2, positive values indicate a relative enhancement of alpha power and negative values indicate a relative suppression of alpha power. The leftmost panel of Figure 2 shows that, as predicted, all four performers exhibited relative alpha suppression in the unison section type (albeit to a small degree for the principal player in the first violin section). Similarly, the middle panel of Figure 2 shows that, as predicted, all four performers exhibited alpha enhancement in the polyphonic section type. Finally, the rightmost panel of Figure 2 shows that, as predicted, in the canon section type, performers in the first violin section exhibited alpha enhancement, whereas performers in the second violin section exhibited alpha suppression.

**Figure 3.**
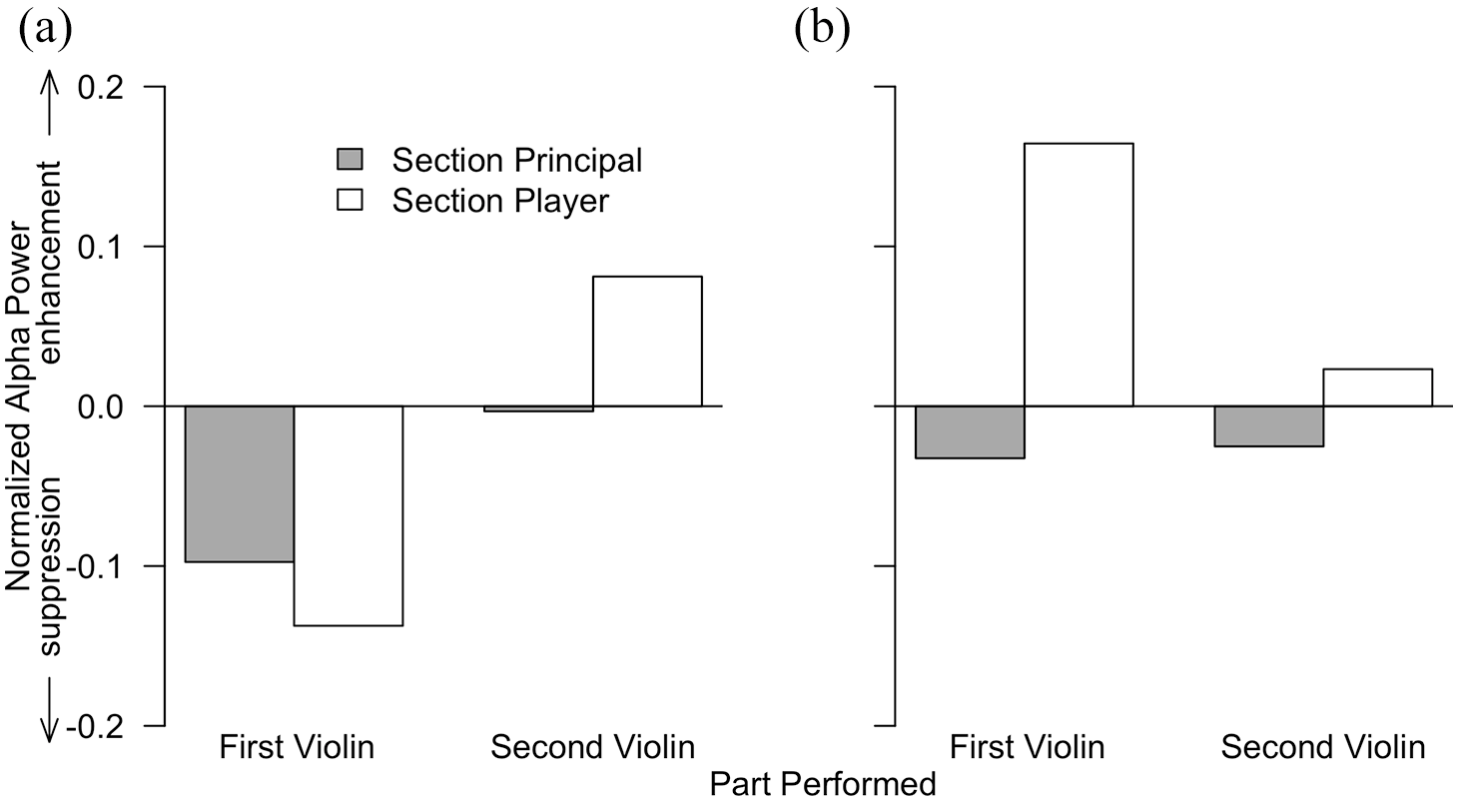
Normalized alpha power for each performer in the first and second violin sections in the homo-divisi (a) and slow homorhythmic (b) section types.

Figure 3 shows the normalized alpha power for each performer in the musical sections for which we did not have *a priori* predictions. The left panel of Figure 3 shows that in the homo-divisi sections, three of the four performers showed alpha suppression and one exhibited alpha enhancement. The right panel of Figure 3 shows that in the slow homorhythmic section, the section principals showed alpha suppression and the section players showed alpha enhancement.

## Discussion

The current study investigated whether the neural underpinnings of self-other integration and distinction that have been identified in previous laboratory experiments would also be evident in a naturalistic music performance setting. The current study yielded three main findings. First, alpha suppression occurred during musical sections that promoted self-other integration by requiring performers to act in a highly congruent manner with one another. Second, alpha enhancement occurred during musical sections that promoted self-other distinction by requiring performers to act in highly incongruent ways from one another. Third, alpha enhancement occurred when performers held a leading role, and alpha suppression occurred when performers held a follower role, during a musical section that required the followers to imitate the musical activity of the leaders at a distance of one bar. As we detail in the following paragraphs, these findings demonstrate that patterns of alpha activity during a real-world performance of a complex musical piece are remarkably consistent with previous findings from laboratory-based experiments that used carefully controlled (and necessarily somewhat artificial) musical materials.

In the current study, violinists displayed a relative suppression of alpha activity when they performed actions that were highly congruent with the actions of other performers in the orchestra. This finding aligns with Novembre and colleagues’ (2016) demonstration of suppressed alpha activity when duetting pianists performed congruent actions. Whereas in Novembre and colleagues’ study, congruent actions took the form of modulating to identical new tempos, in the current study, the violinists’ congruent actions took the form of producing pitches, rhythms, and tempo modulations in unison with each other and the rest of the orchestra. Thus, the current study suggests that Novembre et al.’s (2016) findings generalize to a broader definition of congruent movement and modulation within joint music performance. The current findings are also consistent with previous research showing that people integrate others’ actions into their own action planning and execution processes when they perform congruent actions aimed toward achieving shared goals (Knoblich et al., 2011), and that suppressed alpha activity supports this process of self-other integration (Babiloni et al., 2002; Muthukumaraswamy & Johnson, 2004; Muthukumaraswamy et al., 2004; Naeem et al., 2012; Tognoli et al., 2007).

Violinists also displayed a relative enhancement of alpha activity when they performed actions that were highly incongruent with those of other performers in the orchestra. This finding likewise aligns with Novembre and colleagues’ (2016) demonstration of enhanced alpha activity when duetting pianists performed incongruent actions (i.e., one pianist modulated to a slower tempo while the other modulated to a faster tempo). In the current study, incongruent actions entailed the violinists entering at different times, having different phrasing, dynamics, and notes to the violinist sitting next to them, and being further challenged by contrasting rhythms in the bass and percussion. Thus, the current study again suggests that Novembre et al.’s (2016) findings generalize to a broader definition of incongruent movement and modulation within joint music performance. The current findings are also consistent with the hypothesis that enhanced alpha activity supports performers making themselves distinct from incompatible surrounding activity, possibly to hear their own part, avoid accidentally entraining with others, and/or maintain a sense of autonomous action control (Fairhurst et al., 2019; Keller et al., 2016; Strauß et al., 2014).

During the canon section of the musical performance, both first violinists displayed a relative enhancement of alpha power when taking on a leading role, and both second violinists displayed a relative suppression of alpha power when taking on a follower role. Enhanced alpha power in the leader role is consistent with previous findings of heightened internally oriented processing among leaders in a joint action. For example, leaders show greater reliance on internal models of action timing and increased activity in brain areas associated with internally driven motor processes such as action planning, initiation, and monitoring (Bolt & Loehr, 2021; Chauvigné et al., 2018; Chauvigné & Brown, 2018; Fairhurst et al., 2014; Konvalinka et al., 2014). Our findings of centroparietal alpha suppression (i.e., over sensorimotor regions) among second violinists performing the follower role in the canon section parallel Vanzella et al.’s (2019) findings of increased activation in sensorimotor regions among second violinists performing the follower role during a canonic musical duet. More broadly, suppressed alpha power in the follower role is consistent with the evidence of heightened externally oriented processing among followers in a joint action. For example, followers are more likely to mimic the timing of their partner’s actions and show increased activity in brain areas associated with processing external sensory information (e.g., Chauvigné et al., 2018; Chauvigné & Brown, 2018; Nowicki et al., 2013).

Taken together, the findings from the musical sections for which we had *a priori* predictions provide preliminary evidence that alpha oscillations related to self-other integration and distinction generalize from controlled laboratory settings to a naturalistic performance setting. These findings contribute to ongoing efforts to probe whether neural mechanisms generalize across contexts, as advocated for by researchers who have emphasized a need to investigate neural mechanisms under “real-world” settings (e.g., Kingstone et al., 2008; Shamay-Tsoory & Mendelsohn, 2019) and by proponents of representative design, whereby research questions are investigated across settings that are representative of the contexts in which phenomena of interest arise (e.g., Holleman et al., 2020; Nastase et al., 2020). Although our sample size of four violinists was small and we report descriptive rather than inferential statistics, it is notable that all performers showed patterns of alpha activity that were in line with our predictions. This is particularly noteworthy considering that the musical materials were selected from the orchestra’s existing repertoire rather than composed to fit an experimental design, and we have already highlighted how our findings thus support generalizability from (in)congruency along a single musical dimension (tempo) to (in)congruency along multiple musical dimensions (e.g., pitch, rhythm, phrasing, and dynamics). Our findings also provide preliminary evidence for generalizability from small- to large-group joint actions. Whereas previous research has typically examined alpha activity among pairs or small groups (e.g., Novembre et al., 2016; Vanzella et al., 2019), our findings show that alpha oscillations related to self-other integration and distinction are also evident in a 60-person ensemble. Our findings thus provide empirical support for previous theoretical work suggesting that similar neurocognitive processes might underlie small- and large-scale joint actions (e.g., Pacherie, 2012). Finally, our findings also add to previous research showing that interpersonal coordination mechanisms generalize from simpler coordination tasks such as finger tapping to more complex tasks such as group music performance (Repp & Su, 2013). Here, comparing the findings of the current study to those of previous studies highlights two specific data analysis issues that could point to a need for further research. Because our goal was to align closely with Novembre et al.’s (2016) analysis methods, we calculated *individually defined alpha bands* and examined alpha activity at *centroparietal* electrodes (parameters we chose before beginning the analysis; see Luck & Gaspelin, 2017). Our findings are consistent with findings from tapping studies that examined different portions of the alpha band (e.g., 10.3–11.5 Hz in Tognoli et al., 2007; 10–12 Hz in Naeem et al., 2012). However, our findings differ from those of Konvalinka et al. (2014), who found alpha suppression at frontal electrodes among leaders in a joint tapping task compared to a noninteractive control condition, rather than alpha suppression at centroparietal electrodes among followers. This might have been due to the nature of the conditions employed in their study (Konvalinka et al., 2014), but could also point to differences in alpha activity between tapping compared to canonic music performance. Further research will be needed to clarify how different analysis choices influence patterns of findings and to fully understand the function of frontal versus centroparietal alpha activity during leader–follower interactions.

One potentially interesting difference between our findings and those of previous research is that the mean values of normalized alpha power reported here are somewhat larger than those reported by Novembre et al. (2016). Although more work is needed to establish whether normalized alpha power is reliably larger in ensemble performance in a naturalistic setting compared to duet performance in a laboratory setting, there are several potential explanations for such a pattern if it does bear out in future research. First, this pattern could arise due to a larger ensemble size alone, consistent with, for example, the influence of choir size on singers’ preferred ratios of own-to-others’ volume (Ternström, 1999). Alternatively, this pattern could arise due to increased complexity of coordination when performers have to contend with (in)congruencies across multiple musical dimensions and against multiple partners, as compared to a single musical dimension and a single partner in Novembre et al. (2016). A third possibility is that the larger normalized alpha power in the current study might have resulted from recording a single take without prior rehearsal. This performance context might have pushed the performers into more extreme levels of self-other integration and distinction due to an increased need to fully attend to the actions of their fellow musicians. It also might have minimized noise in the EEG signal that could result from performers employing different coordination strategies over a series of performances. Future research that systematically manipulates ensemble size, coordination complexity, previous practice, and the number of performances could be useful for understanding whether and how these factors influence the power of alpha oscillations related to self-other integration and distinction.

The manipulations just described could also prove useful for uncovering the relationship between performers’ higher-level intentions for self-other integration and distinction and the implementation of self-other integration and distinction at the neural level. For example, the current study might represent a situation that involves a somewhat reduced influence of shared intentions, because the musical piece was performed without immediately prior group rehearsal. Comparing the current study’s findings against those from a performance preceded by extensive group rehearsal could be one way to examine how relatively strong or salient shared intentions might impact neural activity related to self-other integration and distinction. Future research could also systematically examine the influence of performers’ individual intentions for coordination on alpha activity. Here, musical sections like the ones for which we had no *a priori* predictions in the current study could prove useful. The first author’s analysis of the musical score and consultations with other expert string players indicated that there was no clear best coordination strategy for these sections. Rather, these sections might invite different coordination strategies from different performers, which would influence whether they favor self-other integration or distinction. For example, in the homo-divisi section, there were a lot of rests for the violinists, and individual performers might have had different strategies for jumping into the larger ongoing stream of music. Future research could ask performers about their coordination strategies during ambiguous musical sections, and investigate whether these strategies are reflected in patterns of alpha activity. Future research could also track the association between alpha activity and performers’ individual or shared intentions across a series of rehearsals or performances. Such a strategy could provide insight into how relations between higher-level intentions and alpha activity develop and whether or not these relations are stable over time. Together, pursuing these types of research questions could help establish causal relations between performers’ higher-level intentions regarding performance and neural activity reflecting the implementation of those intentions.

Another set of research questions that could be addressed in future research concerns how neural activity related to self-other integration and distinction is influenced by musical expertise, experience performing with specific co-performers, and performers’ roles *within* their instrument section. With respect to musical expertise, we note here that all of the violinists who participated in the current study had many years of performance experience including a considerable amount of ensemble performance experience. Musical training develops communicative strategies to share intentions (Goupil et al., 2021), further develops information parsing in a noisy environment (Coffey et al., 2017; Swaminathan et al., 2015), and increases an individual’s flexibility to others’ musical timing (Scheurich et al., 2018, 2020). Examining neural activity among amateur musicians or as musicians develop their expertise or gain experience performing with others could elucidate the roles of each of these factors in self-other integration and distinction at the neural level. In a similar vein, future research could investigate how performers’ experience playing together with the same set of co-performers might impact alpha activity. Although we did not collect data concerning how long our participants had been performing together with their stand partner or with the orchestra, experience playing together with specific co-performers likely strengthens high-level knowledge about others’ intentions and enhances the ability to predict their actions (Bishop et al., 2019; Ragert et al., 2013; Williamon & Davidson, 2002), which could in turn influence self-other integration and distinction at the neural level. Finally, future research could examine the conditions under which performers’ roles *within* their instrument section (e.g., section principal versus section player) influence self-other integration and distinction. For example, the pattern of alpha activity across performers in the slow homorhythmic section suggests that in this musical section, section principals favored self-other distinction and section players favored self-other integration. Although we do not wish to draw strong conclusions from this finding because it was not predicted *a priori*, it does suggest that investigating the influence of performers’ roles could be a fruitful avenue for future work.

As the preceding paragraphs make clear, a number of interesting questions remain to be explored in future research. Continued work toward understanding brain activity related to self-other integration and distinction could yield useful applications across performance, educational, and therapeutic settings. For example, in performance settings, alpha oscillations could provide an index of whether performers are prioritizing taking a leading role or integrating with other group members. Rehearsal strategies could then be selected to focus on enhancing performers’ strategies or reversing them, depending on performance goals. In other words, performers’ brain activity could act as a prompt for problem-solving behavior alongside other prompts used by teachers and co-performers (cf. Roesler, 2017, 2022). Another potential application for performance settings could be to examine whether neural activity reflecting self-other integration and distinction extends to audience members who engage with the musical performance (e.g., by moving or singing along with the music). Such research could potentially elucidate the conditions under which mutual coordination occurs between performers and audience members (cf. Brand et al., 2012). Alpha oscillations could also be used as an index of self-other integration and distinction in music therapy settings, where baseline activity could be measured and therapeutic techniques could be selected to emphasize self-related or relational processes and experiences, depending on therapeutic goals (cf. Maratos et al., 2011). A similar application could be envisioned for music education settings, where alpha oscillations could also provide a useful index to shape educators’ strategies for teaching and learning. At a broader level, findings from this type of research could illustrate the value of using music performance, education, or therapy to enhance leadership- and teamwork-related processes that apply across any environment in which people must cooperate within a group.

## Conclusion

In sum, findings from the current study further our understanding of the neural underpinnings of self-other integration and distinction by demonstrating that patterns of alpha activity previously reported in small-scale joint actions under strictly controlled laboratory conditions are also evident in a large-scale joint action taking place in a natural performance environment. These findings suggest interesting avenues for future research, such as how ensemble size and performers’ strategy choices influence self-other integration and distinction and concomitant patterns of alpha activity. They also contribute to a growing body of research that investigates brain activity during ecologically valid interpersonal interactions and its potential implications for applied settings such as music performance, education, and therapy.

## Notes

1. The first author has over 30 years of performance experience and over 20 years of compositional and musical analysis experience. Analytical decisions were further confirmed by consultation with two professional string player colleagues of the first author.

2. String players are seated in pairs and share a music stand. Stand partners often play identical musical material. However, stand partners divide the musical material between them when there are two staves for each violin part as shown in Figure 1(b) (i.e., two parts labeled “Vln. I” and two parts labeled “Vln. II”) or when the score is marked *divisi* as shown in Figure 1(e). Our participants were not stand partners with each other, but they were still expected to be affected by incongruence between their own score indications and those of their (nonrecorded) stand partner.

3. Together, the five musical section types comprise all of the violinists’ performed material in the score. Bars consisting entirely of rests were not analyzed.

4. To ensure that EEG and video data were properly aligned, we confirmed that the time elapsed between eye-closing peaks in the EEG data matched the time elapsed between eye-closings in the video data (first visible frame), for all 12 blinks for each participant when possible.

5. The Savitzky–Golay smoothing used in the restingIAF function allows for a finer frequency resolution of ~0.24 Hz.
